# Temporal dynamics of serum S100β as a prognostic indicator in aneurysmal subarachnoid hemorrhage: A prospective cohort study including comparison of clipping and endovascular treatment

**DOI:** 10.1016/j.bas.2026.106141

**Published:** 2026-06-27

**Authors:** P. Geiger, S.A. Treichl, W. Ho, C. Preuss-Hernandez, D. Pinggera, C. Thomé, O. Petr

**Affiliations:** a Department of Neurosurgery, Medical University Innsbruck, Tyrol, Austria; b Department of Neurosurgery, Charles University Hospital, Prague, Czech Republic

**Keywords:** S100-beta, Aneurysmal subarachnoid hemorrhage, Prognostic biomarker, Serum dynamics, Surgical clipping, Endovascular coiling

## Abstract

**Introduction:**

S100β is an astrocyte-derived calcium-binding protein with prognostic utility after brain injury. Its temporal profile in aneurysmal subarachnoid hemorrhage (aSAH) and the influence of peri-procedural factors on serum levels remain incompletely characterized.

**Research question:**

(1) Are serial serum S100β concentrations associated with six-month functional outcome after aSAH? (2) Do S100β trajectories differ between surgical clipping and endovascular treatment, compared with an elective unruptured aneurysm cohort as a procedural reference?

**Methods:**

This prospective single-center cohort study enrolled 100 adults (50 aSAH; 50 elective unruptured aneurysm treatments). Venous blood was sampled pre-intervention and repeatedly through day 10. S100β was measured using the Elecsys® S100 assay (Roche). Six-month outcome was assessed with the Glasgow Outcome Scale, dichotomized as favorable (4–5) vs. unfavorable (1–3). Nonparametric comparisons and logistic regression evaluated associations between S100β, treatment modality, and outcome.

**Results:**

Baseline S100β was higher in aSAH than elective controls (median 0.19 vs. 0.08 μg/L; p < 0.001). Surgical clipping was associated with higher early post-procedural S100β than endovascular treatment through day 2, with no clear difference thereafter. Higher pre- and early post-intervention S100β values were associated with unfavorable six-month outcome. Treatment modality itself was not associated with outcome.

**Conclusion:**

Serial S100β elevations during acute aSAH were associated with unfavorable long-term outcome, supporting S100β as a candidate prognostic marker rather than a validated standalone biomarker. Early post-procedural S100β elevations after surgical clipping were transient and may reflect peri-procedural glial activation, baseline severity differences, and other confounding factors. These exploratory findings require confirmation in larger, adequately adjusted multicenter cohorts before clinical prognostic implementation.

## Highlights

• Serial S100-beta measurements demonstrate prognostic utility in aSAH versus elective aneurysm treatment in the postoperative phase.
• Elevated S100-beta in aSAH show predictive value for unfavorable clinical outcome.
• Surgical clipping induces higher S100-beta protein in the serum compared to endovascular treatment, with no difference in six-month functional outcome in relation to endovascular treatment.
• Transient, clipping-associated S100-beta elevation reflects reversible astrocytic activation rather than irreversible damage.

## 1 Introduction

Aneurysmal subarachnoid hemorrhage (aSAH) is a devastating cerebrovascular condition with significant morbidity and mortality, often leaving survivors with severe neurological deficits or lifelong dependency (Lai and Du, 2016). Despite advances in neurosurgical and critical care, predicting long-term outcome in aSAH remains a clinical challenge. Reliable prognostic tools are essential to guide treatment decisions, monitor disease progression, and optimize clinical long-term outcome. Biomarkers reflecting the extent of brain injury and recovery processes have emerged as promising candidates for this purpose (Kellermann et al., 2016a; Sanchez-Peña et al., 2008).

S100β, a calcium-binding protein predominantly expressed in astrocytes, has gained attention as a biomarker for brain injury. Initially studied in the context of traumatic brain injury (TBI), it has been shown to correlate with the degree of astrocytic activation and neuronal damage. Its release into the bloodstream or cerebrospinal fluid (CSF) occurs following blood-brain barrier disruption or glial injury, making it a potential indicator of pathological processes in aSAH. Beyond its intracellular roles in calcium homeostasis, enzyme regulation, and cytoskeletal dynamics, extracellular S100β exerts paracrine effects that include neuroprotection, inhibition of apoptosis, and promotion of neuroregeneration. However, at elevated concentrations, S100β may exacerbate neuroinflammation and secondary brain injury (Balança et al., 2020; Zhou et al., 2022; Kedziora et al., 2020).

In aSAH, the dynamic changes in S100β levels reflect the complex interplay between primary injury from aneurysm rupture and secondary processes such as delayed cerebral ischemia (DCI). Previous studies have demonstrated that elevated S100β levels are associated with poor neurological outcome and complications like DCI.

However, three clinically relevant aspects remain incompletely characterized: first, the temporal profile of serum S100β from the pre-interventional phase through the early post-treatment period; second, the extent to which S100β elevations reflect hemorrhage-related injury versus procedure-related biomarker release; and third, whether the interpretation of S100β differs after microsurgical clipping and endovascular treatment.

Additionally, the prognostic utility of serum versus CSF S100β levels and their relationship to clinical outcome warrants further investigation (Kedziora et al., 2020; Aineskog et al., 2022). The primary aim of this prospective cohort study was therefore to characterize serial serum S100β dynamics in aSAH and to examine their association with six-month functional outcome. As secondary exploratory objectives, we compared S100β trajectories between clipping and endovascular treatment and included an elective unruptured aneurysm cohort as a procedural reference group. This design was intended to provide temporal and mechanistic context for S100β interpretation, rather than to establish a validated prognostic model or infer causal treatment effects.”

## 2 Methods

Data were collected prospectively between April 2016 and June 2017. Treatment protocols, S100β assay methodology (including reagent lot stability and analyzer calibration), and six-month outcome assessment procedures remained consistent throughout the entire enrollment period. No changes to institutional neurovascular treatment guidelines or laboratory infrastructure occurred during this interval.

This prospective single-center cohort study was conducted at a tertiary care center between April 2016 and June 2017. The study adhered to the Declaration of Helsinki and was approved by the institutional ethics committee. Written informed consent was obtained from all participants or their legal representatives.

This study comprised a cohort of 100 patients: one consisting of patients with aSAH caused by spontaneous rupture of intracranial aneurysms and a second group receiving elective treatment for incidental unruptured intracranial aneurysms.

Inclusion criteria required patients to have confirmed intracranial aneurysms based on cranial computed tomography (CCT) and angiographic studies. Exclusion criteria included traumatic SAH, cerebrovascular malformations, pregnancy, and insufficient data availability.

Baseline neurological status was assessed using the Hunt and Hess grading scale. Radiological evaluations included initial CCT scans and angiographic studies to confirm the diagnosis and guide therapeutic decision-making. The modified Fisher scale was used to classify the extent of hemorrhage in SAH patients. Treatment allocation – surgical clipping versus endovascular coiling – was determined by the treating neurovascular team on the basis of aneurysm morphology, neck anatomy, location, and clinical presentation grade, consistent with institutional and international guidelines. Patients were not randomized to treatment modality. As a consequence, patients assigned to surgical clipping presented with slightly milder hemorrhagic grades on average. This non-randomized allocation introduces confounding by indication that must be considered when interpreting between-group biomarker comparisons; such comparisons are therefore presented as descriptive and hypothesis-generating rather than as evidence of independent treatment effects.

The elective cohort was not intended as a clinically equivalent comparator to the aSAH population, as these groups differ fundamentally in disease biology, indication for treatment, perioperative course, and intensive care exposure. Rather, elective patients were enrolled to provide a procedural frame of reference: by quantifying the S100β response attributable to the intervention itself – in the absence of hemorrhagic injury – this cohort allows the hemorrhage-specific biomarker signal in aSAH patients to be contextualized. Direct between-cohort comparisons are interpreted with this structural limitation in mind.

Serial venous blood samples for serum S100β analysis were collected at predefined time points.

- Pre-intervention (baseline)
- Intra-intervention at timepoint 0
- Post-intervention at 6 h, 12 h, and daily up to 10 days

CSF samples were collected only in aSAH patients when clinically available. This included intraoperative CSF sampling during aneurysm treatment (when feasible) and/or sampling via an external ventricular drain (EVD) when present. S100β concentrations in serum and CSF were quantified using the Elecsys® S100 assay (Roche Diagnostics), with reference values established for analysis.

Functional outcome was assessed at six months post-ictus using the Glasgow Outcome Scale (GOS), dichotomized into favorable (GOS 4–5: good recovery or moderate disability) and unfavorable (GOS 1–3: severe disability, vegetative state, or death). Six-month GOS was assessed by routine clinical follow-up. Outcome assessment was not blinded to treatment modality or S100β values.

Statistical analyses were performed using Jamovi (Version 2.5) and R (Version 4.3). Because S100β concentrations were non-normally distributed at all time points, nonparametric methods were used throughout. Between-group comparisons at individual time points were performed using the Mann–Whitney *U* test; effect size was quantified using the biserial rank correlation coefficient (r). For the primary exploratory prognostic analysis, Mann–Whitney U tests were applied at each serum time point to compare S100β concentrations between patients with favorable (GOS 4–5) and unfavorable (GOS 1–3) six-month outcomes, stratified by treatment modality where appropriate (clipped aSAH cohort: n = 30; coiled aSAH cohort: n = 20). For the treatment-modality comparison, Mann–Whitney U tests were applied at each serum time point to compare S100β concentrations between surgically clipped (n = 30) and endovascularly treated/coiled (n = 20) patients within the aSAH cohort. In addition, two univariable exploratory logistic regression models were constructed. The first model assessed the unadjusted association between treatment modality (clipping vs. coiling, coded as a binary predictor) and unfavorable six-month GOS outcome in the aSAH cohort. The second model, performed within the clipped aSAH subgroup, examined the unadjusted association between pre-intervention serum S100β and six-month outcome. These regression analyses were retained to provide transparent exploratory estimates; however, they were not intended to establish independent predictive value or causal treatment effects. Given the limited number of outcome events and the small subgroup sizes, multivariable adjustment for established prognostic factors such as clinical SAH severity, radiographic hemorrhage burden, aneurysm characteristics, age, and other relevant covariates was not performed, as such models would be at high risk of overfitting and statistical instability. Accordingly, all regression results should be interpreted as descriptive, exploratory, and hypothesis-generating only. Given the multiple pairwise comparisons performed across time points, no formal correction for multiplicity was applied; all time-course findings are therefore considered exploratory. Analyses were based on available cases at each time point; sample sizes at each interval are reported in the Results.

## 3 Results

The study enrolled 100 patients divided into two cohorts: 50 patients with aSAH and 50 undergoing elective treatment for incidental intracranial aneurysms. Participants had a mean age of 54.7 years (range: 29–81), with comparable distributions between groups (aSAH: 53.8 years; elective: 55.6 years). Both cohorts exhibited a female predominance (male-to-female ratio: 1:1.9 overall; aSAH: 1:2.3; elective: 1:1.6). Comorbidities such as arterial hypertension (48% vs. 21.8%) and nicotine abuse (35% vs. 17.9%) were more prevalent in the aSAH group. Aneurysms were predominantly located in the anterior circulation (aSAH: 86.0%; elective: 84.0%). Patients were treated either by surgical clipping or by endovascular treatment; because modality distributions differ between the aSAH and elective cohorts, modality counts and percentages are reported separately for each cohort below (and should not be interpreted from the pooled 100-patient denominator). Perioperative complications were more frequent in the aSAH group (38% vs. 6%). Hospitalization duration was significantly longer for aSAH patients (mean 29.1 days vs. 11.5 days), with intensive care unit stays averaging 16.2 days versus 1.1 days in the elective group. The mean GOS at discharge was 4.4 (1.2), mean GOS at 6 months was 4.5 (1.1). When SAH was present, mean GOS at discharge was 3.7 (1.5) and 4.0 (1.5) at 6 months follow-up. In incidental cases, mean GOS at discharge and after 6 months was 4.9 (0.3). Demographic data are shown in Table 1.

**Table 1:** Summarized patient data.

	Overall (N = 100)
**Age**	
Mean (SD)	54.7 (11.2)
Range	29.0 - 81.0
**Sex**	
Male	34 (34.0%)
Female	66 (66.0%)
**Height [cm]**	
Mean (SD)	169.2 (7.9)
Range	153.0 - 188.0
**Weight [kg]**	
Mean (SD)	73.2 (19.4)
Range	43.0 - 170.0
**Nicotine abuse**	
N/A	16
No	50 (59.5%)
Yes	34 (40.5%)
**Arterial Hypertension**	
N/A	3
No	49 (50.5%)
Yes	48 (49.5%)
**Positive familial history for cerebral aneurysms**	
N/A	2
No	89 (90.8%)
Yes	9 (9.2%)
**OAC/PAI**	
No	74 (74.0%)
Yes	26 (26.0%)
**SAH**	
No	50 (50.0%)
Yes	50 (50.0%)
**H&H**	
Incidental	50 (50%)
1	14 (14.0%)
2	6 (6.0%)
3	16 (16.0%)
4	9 (9.0%)
5	5 (5.0%)
**WFNS**	
1	73 (73.0%)
2	2 (2.0%)
3	10 (10.0%)
4	4 (4.0%)
5	11 (11.0%)
**Fisher**	
N/A	7
0	18 (19.4%)
1	27 (29.0%)
2	10 (10.8%)
3	11 (11.8%)
4	26 (28.0%)
5	1 (1.1%)

### 3.1 Stratified analysis

#### 3.1.1 S100β levels in clipped vs. coiled aneurysm patients

**Fig. 1:**
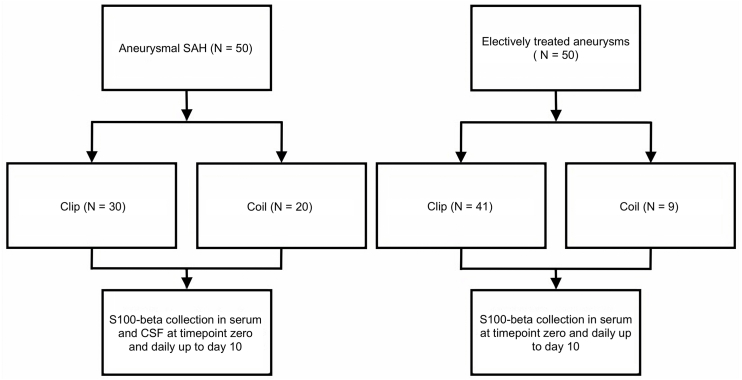
Flow-Chart of group splitting and S100β data acquisition.

**Fig. 2:**
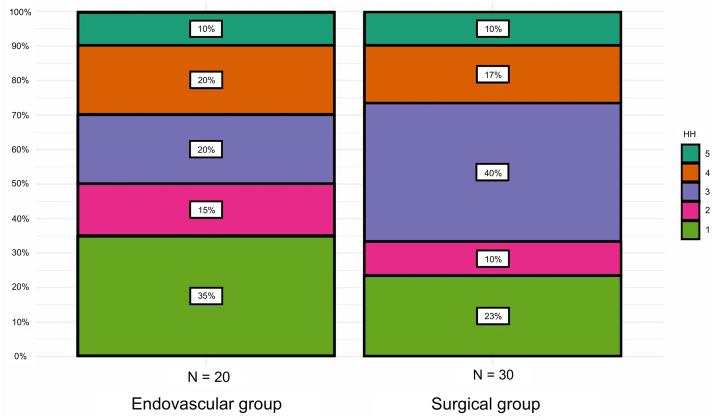
Differences in Hunt and Hess (HH) grades in surgical vs. endovascular SAH groups.

Analysis of serum S100β concentrations stratified by treatment modality suggested differences during the early post-procedural phase between patients treated with surgical clipping (n = 30 within the aSAH cohort) and endovascular treatment/coiling (n = 20 within the aSAH cohort) (see Fig. 1). Hunt and Hess grade distribution is portrayed in Fig. 2. Pre-intervention S100β levels did not differ significantly between modalities (p = 0.32). Clipped patients exhibited higher serum S100β concentrations at several consecutive early time points: timepoint 0 (p = 0.03), 6 h (p < 0.01), 12 h (p < 0.01), day 1 (p < 0.01), and day 2 (p = 0.02). From day 3 onward, differences diminished, with no significant differences observed at day 3 (p = 0.27) or day 5 (p = 0.86). By day 10, S100β levels showed a trend toward convergence (p = 0.08).

**Data completeness:** Sample availability varied by time point. Serum S100β was available (n with measured sample out of N = 100) at pre-intervention: 93; timepoint 0: 93; 6 h: 80; 12 h: 53; day 1: 92; day 2: 96; day 3: 94; day 4: 92; day 5: 86; day 6: 78; day 7: 70; day 8: 55; day 9: 51; day 10: 41. CSF S100β was obtained only in aSAH patients (N = 50) when clinically available (primarily via EVD; EVD present in 26/50); CSF availability at timepoint 0 could exceed the EVD count because intraoperative CSF was also obtained in some patients without EVD. CSF S100β was available (n with measured sample out of N = 50 aSAH patients) at pre-intervention: 15; timepoint 0: 36; 6 h: 11; 12 h: 6; day 1: 15; day 2: 17; day 3: 14; day 4: 15; day 5: 15; day 6: 17; day 7: 16; day 8: 15; day 9: 16; day 10: 14.

#### 3.1.2 S100β levels in SAH vs. incidental cases

**Fig. 3:**
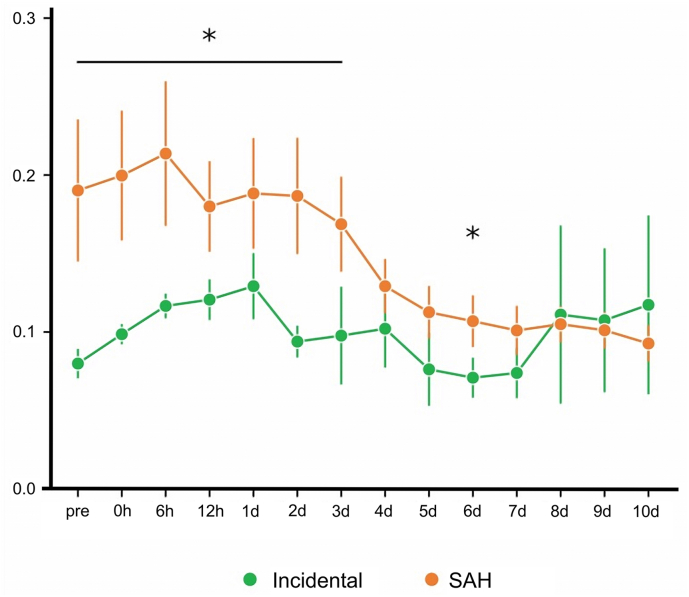
Differences in S100 β in serum in SAH patients vs. elective cases. Serum levels measured in μg/L. Means were calculated for comparability.

**Fig. 4:**
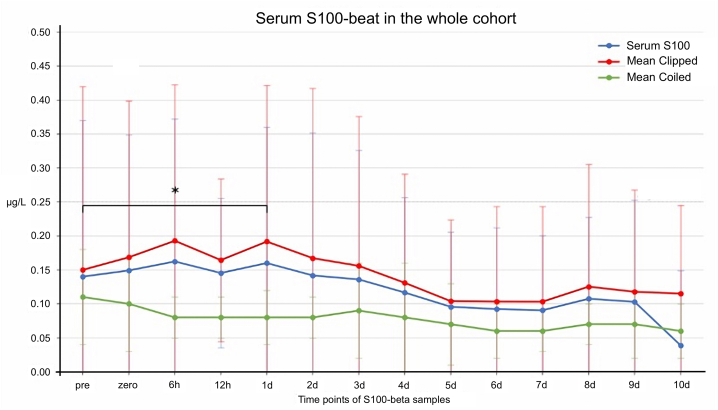
Serum S100β concentrations over time in the overall cohort and stratified by treatment modality. Values are shown in μg/L.

Patients with aSAH exhibited consistently higher serum S100β concentrations compared to those with incidental aneurysms throughout the study period. Pre-intervention baseline S100β levels were significantly higher in the aSAH group (0.19 μg/L versus 0.08 μg/L, p < 0.001). Post-intervention differences reached statistical significance at multiple intervals during the acute phase, including levels in serum at timepoint 0 (p = 0.04), serum at day 2 (p = 0.01), and serum at day 5 (p = 0.01). In contrast, no significant differences were observed during the later recovery phase. Data for SAH-patients is visualized in Fig. 3. S100-beta data for the whole cohort is displayed in Fig. 4.

#### 3.1.3 Serum and CSF S100β levels in SAH and clipped aneurysms in relation to outcome

Pre-intervention serum S100β levels were higher in patients with unfavorable outcome (p = 0.003, biserial rank correlation = 0.841), suggesting that baseline concentrations capture clinically relevant information related to the initial hemorrhagic insult and early brain injury before therapeutic intervention. This association should not be interpreted as independent prognostic validation, because the present cohort was not sufficiently powered to adjust simultaneously for established clinical and radiographic severity variables. Postoperative elevations persisted in the poor outcome group at serum timepoint 0 (p = 0.006), serum at hour 6 (p = 0.012), serum at day 1 (p < 0.001), and serum at day 2 (p = 0.004), with the strongest separation between outcome groups observed at day 1 (biserial rank correlation = 0.911). Associations attenuated over time; by day 10 only a weak, non-significant association remained (p = 0.076).

Cerebrospinal fluid S100β levels demonstrated less consistent prognostic utility compared to serum. Despite higher mean concentrations in the poor outcome group (e.g., CSF zero: 78.6 μg/L vs. 19.31 μg/L in the good outcome group), statistical significance was absent at most timepoints (p = 0.19 at CSF zero; p = 0.91 at CSF 10d). This discrepancy likely stems from smaller CSF sample sizes and greater variability, as evidenced by overlapping interquartile ranges. Notably, large effect sizes were observed at select intervals, such as CSF 2d (biserial rank correlation = 1.00; p = 0.056), suggesting pathological relevance that warrants further investigation. At later stages (e.g., CSF 6d, CSF 9d), mean CSF S100β remained elevated in the poor outcome group but lacked statistical power for definitive conclusions.

Clipping was not associated with unfavorable outcome after 6 months in the overall cohort (p = 0.977, 95% CI [0.25 to 3.34]) or in the aSAH subgroup (p = 0.29, 95% CI [0.1048 to 1.833]).

#### 3.1.4 Serum and CSF S100β in coiled SAH patients in relation to outcome

The endovascular SAH cohort comprised 20 cases. In coiled SAH patients, serum S100β levels showed no consistent differences between good and poor outcome groups across most time points. Pre-intervention levels showed a non-significant trend toward higher values in the poor outcome group (p = 0.362, biserial rank correlation = 0.333). Early postoperative timepoints (timepoint 0, 6 h, day 1) showed moderate effect sizes but did not reach statistical significance. An isolated difference was observed at day 9 (p = 0.008, biserial rank correlation = 0.950).

**Fig. 5:**
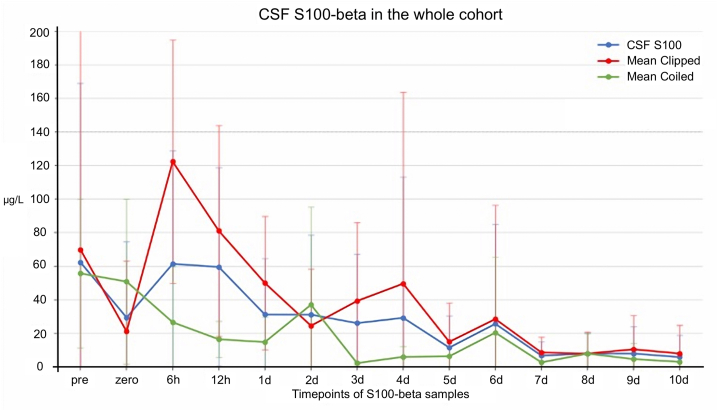
CSF S100β concentrations over time in available aSAH samples. Greater variability reflects limited and clinically driven CSF sampling. Values are shown in μg/L.

CSF S100β levels similarly showed no significant differences between outcome groups across most timepoints. Pre-intervention CSF concentrations were comparable between groups (p = 0.857), and postoperative measurements in CSF at timepoint 0 (p = 0.914) and at day 10 (p = 1.000) remained nonsignificant. Descriptive statistics revealed elevated mean CSF S100β concentrations in the poor outcome group (e.g., CSF zero: 78.6 μg/L vs. 19.31 μg/L in the good outcome group), however, the limited and clinically driven availability of CSF samples, together with overlapping distributions, precluded definitive subgroup-level conclusions. CSF S100-beta levels are shown in Fig. 5.

#### 3.1.5 Treatment modality and neurological outcome

**Fig. 6:**
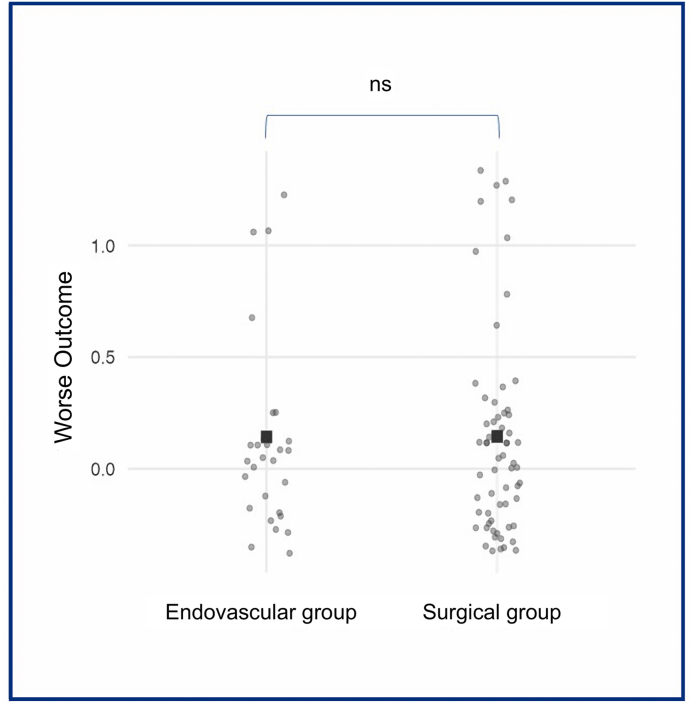
Exploratory univariable analysis of treatment modality and six-month functional outcome in the aSAH cohort. Surgical clipping was not statistically significantly associated with unfavorable six-month outcome; however, this unadjusted finding should be interpreted as descriptive and hypothesis-generating only.

Logistic regression analysis revealed that clipping was not significantly associated with unfavorable outcome at six months in the whole cohort (p = 0.977). This absence of association persisted when analysis was restricted to the aSAH group (p = 0.29), indicating that treatment modality per se did not independently predict long-term functional outcome (see Fig. 6).

Of note, exploratory univariable logistic regression was performed to provide transparent descriptive estimates. Treatment modality was not significantly associated with unfavorable six-month outcome in the aSAH cohort; however, because this model was unadjusted and the cohort was not powered for multivariable adjustment, this finding should not be interpreted as evidence of treatment equivalence or absence of a causal treatment effect. Similarly, the univariable association between pre-intervention serum S100β and outcome in the clipped aSAH subgroup should be interpreted as hypothesis-generating and not as independent prognostic validation.

## 4 Discussion

In this prospective single-center cohort study, serially measured serum S100β concentrations were associated with six-month functional outcome in patients with aneurysmal subarachnoid hemorrhage. Patients with unfavorable outcome showed higher pre-interventional and early post-interventional S100β concentrations, indicating that acute-phase S100β reflects clinically relevant aspects of early brain injury and disease severity. Importantly, the present study should be interpreted as an exploratory temporal biomarker-characterization study rather than as definitive prognostic validation.

The findings are consistent with prior literature linking S100β to outcome after aSAH, while adding detailed peri-interventional time-course data and a procedural reference cohort of elective unruptured aneurysm treatments. Larger multicenter cohorts with sufficient events for multivariable adjustment are required before serum S100β can be recommended as a standalone clinical prognostic tool (Marchi et al., 2003).

Our study demonstrates that serum S100β concentrations measured from pre-intervention through the acute postoperative phase were associated with unfavorable neurological outcome at six months after aSAH. Pre-intervention S100β levels were significantly elevated in patients with poor outcome (p = 0.003, biserial rank correlation = 0.841), indicating that baseline concentrations already reflect substantial prognostic information capturing the severity of the initial hemorrhagic insult before any therapeutic intervention occurs. This observation aligns with systematic reviews demonstrating that higher serum S100β levels correlate with worse GOS outcomes, supporting S100β as a promising prognostic candidate in the aSAH population (Lai and Du, 2016). Whether this association reflects an independent biomarker effect or is primarily mediated by initial hemorrhage severity – with higher-grade aSAH driving both greater S100β release and worse prognosis – could not be fully disentangled in the present cohort due to the limited number of outcome events relative to candidate covariates; this remains an important question for future adequately powered studies. Taken together, these findings support S100β as an accessible biomarker that captures the biological severity of early brain injury after aSAH, while not yet establishing independent prognostic value beyond established clinical grading systems.

The temporal evolution of S100β concentrations reveals insights into the pathophysiology of secondary brain injury following aSAH. The strongest prognostic correlation was observed at day one postoperatively (p < 0.001, biserial rank correlation = 0.911), indicating that S100β dynamics during the first 24 h following intervention most robustly reflect long-term functional trajectories. This acute-phase elevation likely reflects the compounded effects of the primary hemorrhagic injury, blood-brain barrier disruption, and evolving neuroinflammatory cascades. Significant differences between outcome groups persisted through the subacute period at day three, though these associations progressively attenuated during the later recovery phase, consistent with temporal resolution of acute neuroinflammatory processes in patients following favorable trajectories (Kellermann et al., 2016a).

The observed biphasic pattern of S100β elevation – peaking acutely post-intervention and declining thereafter – is consistent with distinct injury mechanisms in aSAH. The pre-intervention elevation (i.e., 0.19 μg/L vs. 0.08 μg/L in elective patients; p < 0.001) reflects the immediate astrocytic response to hemorrhage-induced neuronal injury, in line with prior studies linking S100β to blood-brain barrier disruption and cytotoxic edema. The sharper rise in aSAH patients immediately after intervention (peak: 0.215 μg/L at 6 h) likely reflects compounded effects from both the initial hemorrhage and surgical manipulation, whereas the elective group's delayed peak (0.13 μg/L at 1 day) is consistent with iatrogenic glial activation from aneurysm occlusion. A study by Kellermann et al. also found a spike in serum and CSF S100β levels after brain injury and correlated their findings with a serum to CSF S100β ratio (Kellermann et al., 2016b). The sustained elevation of S100β in aSAH patients during the first five postoperative days contrasts with the rapid normalization in elective patients, mirroring the prolonged neuroinflammatory milieu characteristic of aSAH. These findings align with Sanchez-Peña et al. who identified mean 15-day S100β levels as predictive of 12-month outcome (Sanchez-Peña et al., 2008).

Serum S100β levels in the acute phase were higher in patients treated by surgical clipping than in those treated by endovascular coiling. This finding is biologically plausible, but its interpretation requires caution. Treatment allocation in this cohort was non-randomized and governed by aneurysm morphology, location, and clinical presentation grade. Accordingly, the observed between-group difference in S100β dynamics may reflect this baseline imbalance rather than a direct causal effect of the surgical approach. The claim that pre-intervention S100β levels showed no significant differences between treatment groups is consistent with this interpretation, though it does not exclude residual confounding. The between-group comparison should therefore be regarded as descriptive and hypothesis-generating; disentangling treatment-specific from severity-driven biomarker effects would require propensity-score adjusted analyses or prospective randomized designs. Critically, our analysis showed no significant effect on clinical outcome at six months in the surgical group overall or in the aSAH subgroup, which is reassuring with respect to the clinical relevance of any procedure-induced biomarker elevation.

Our observation that elevated S100β levels show prognostic associations with poor clinical outcome in aSAH is supported by multiple studies (Balança et al., 2020). Zhou et al. found that serum S100β and neurofilament light chain (NfL) levels were significantly associated with Hunt-Hess grade, WFNS, and Fisher grade, with higher values correlating with poorer prognosis (Zhou et al., 2022). Similarly, Kedziora et al. demonstrated that peripheral S100β level serves as a useful biomarker for mortality prediction with an area under the curve of 0.825 (Kedziora et al., 2020).

While serum S100β showed consistent prognostic associations, CSF measurements demonstrated limited consistency despite large effect sizes (e.g., CSF 2d: p = 0.056). This discrepancy likely reflects CSF's narrower temporal window of relevance and the technical challenges inherent to serial sampling, as CSF collection was restricted to patients with external ventricular drains in situ — a structural limitation that renders direct comparison with the serum cohort inherently unbalanced. The higher mean CSF concentrations in poor-outcome patients (i.e., CSF zero: 78.6 μg/L vs. 19.31 μg/L) suggest that CSF S100β may capture focal injury patterns not reflected by systemic serum levels, though larger cohorts with systematic CSF access are needed to validate this hypothesis. Marchi et al. suggested that serum S100β represents a marker of blood-brain barrier integrity rather than direct neuronal damage, which may explain why the substantial spike in CSF S100β relative to serum S100β resembles a bleed-through effect, further underscoring the complementary value of both measurement compartments (Aineskog et al., 2022; Marchi et al., 2003).

The mechanistic basis for clipping-associated S100β elevation is multifactorial and plausibly attributable to the invasive nature of open neurosurgical procedures. Surgical clipping necessitates craniotomy, brain retraction, microdissection through perianeurysmal tissues, temporary vessel occlusion, and mechanical manipulation of surrounding parenchyma. Brain retraction represents a well-characterized mechanism of iatrogenic injury, producing focal tissue compression, ischemic stress, and disruption of cellular membrane integrity that collectively promote biomarker release. Konya and colleagues documented brain retraction injury in 15.2% of patients undergoing elective aneurysm clipping, with associated risks of postoperative focal neurological deficits (Konya et al., 2022; Zhu et al., 2022). This aligns with meta-analysis findings by Zhu et al. who reported that clipping was associated with higher rates of vasospasm at discharge compared to endovascular treatment, potentially reflecting procedural trauma (Zhu et al., 2022). We therefore interpret the transient post-procedural S100β elevation observed after surgical clipping as mechanistically compatible with peri-procedural glial activation, blood-brain barrier perturbation, and limited tissue manipulation-related biomarker release. Because this interpretation is based on biomarker dynamics rather than direct histopathological or advanced neuroimaging confirmation, it should be regarded as a biologically plausible hypothesis rather than definitive proof of reversible astrocytic activation. Notably, the association between higher acute-phase S100β values and unfavorable outcome does not contradict the hypothesis that a component of the early clipping-associated S100β increase may reflect reversible peri-procedural astrocytic activation. These observations address different biological signals. The outcome-associated S100β elevation was already present before intervention and therefore most likely reflects the severity of the initial hemorrhagic insult and early brain injury. By contrast, the modality-associated increase after clipping was transient, most evident during the early post-procedural window, and converged thereafter. Thus, the clipping-related component should be interpreted cautiously as a possible procedure-related biomarker signal superimposed on hemorrhage-related injury, not as evidence of durable parenchymal damage or as an explanation for unfavorable outcome.

The novelty of the present study does not lie in demonstrating for the first time that S100β is associated with outcome after aSAH, as this relationship has been suggested previously. Rather, the incremental contribution lies in the standardized, prospective, serial sampling framework, which captures S100β dynamics from the pre-interventional phase through day 10; the inclusion of an elective unruptured aneurysm cohort as a procedural reference; and the parallel examination of clipping- and endovascular-treatment-associated trajectories within the same institutional workflow. This design allows a more nuanced interpretation of S100β as a timing-dependent marker influenced by hemorrhage severity, peri-interventional factors, and treatment context. The study therefore complements prior prognostic literature by emphasizing when and under which procedural circumstances S100β measurements may be most informative.

In summary, in our prospective single-center cohort, higher acute-phase serum S100β concentrations were associated with unfavorable six-month functional outcome after aSAH, supporting S100β as a potentially clinically informative candidate marker in this population. Early post-procedural serum S100β levels were higher in patients treated with surgical clipping than in those treated endovascularly; however, given the non-randomized treatment allocation and likely differences in baseline demographics, these modality-related elevations should not be interpreted as direct evidence of procedure-induced parenchymal injury. The convergence of S100β trajectories between clipping and endovascular treatment by approximately day 3, together with the absence of an independent association between treatment modality and six-month outcome, indicates that early biomarker peaks primarily reflect underlying disease burden and peri-interventional factors rather than durable modality-specific damage. The exploratory logistic regression comparing clipping versus coiling in relation to six-month outcome was included to complement the descriptive biomarker analyses by examining whether the observed modality-related S100β differences translated into a detectable signal at the level of functional outcome. Given the limited sample size and absence of multivariable adjustment, these models were not intended to provide causal inference but rather to test whether any large, unadjusted effect of treatment modality on outcome could be detected in this cohort. The lack of a significant association therefore supports the conclusion that the observed early S100β differences are not accompanied by a marked, unadjusted outcome penalty of clipping within the constraints of this study design.

Our findings extend prior work on S100β after aSAH by characterizing detailed temporal serum profiles in parallel elective unruptured aneurysm and ruptured cohorts and by directly comparing clipping and endovascular repair within the same standardized sampling framework. In doing so, this study highlights that the timing and dynamics of S100β release are critical for interpretation and that single, opportunistic measurements are unlikely to provide robust risk stratification. Future multicenter studies with larger sample sizes, harmonized pre-analytical procedures, and adjusted or randomized comparisons are warranted to (1) define clinically actionable S100β thresholds for early risk stratification, (2) clarify the incremental prognostic value of S100β over established clinical and radiographic severity scales, and (3) explore whether integration of S100β into multimodal monitoring paradigms can meaningfully inform treatment decisions and long-term outcome prediction after aSAH.

## 5 Limitations

Several limitations of this study warrant consideration. Most importantly, the study was not powered to determine whether S100β adds independent prognostic value beyond established clinical and radiographic severity variables, nor to support adjusted causal comparisons between clipping and endovascular treatment. The treatment allocation was non-randomized and determined by clinical and anatomical factors, resulting in systematic baseline differences between the surgical and endovascular groups. This confounding by indication limits the interpretability of between-group biomarker comparisons and precludes causal inference regarding treatment-specific effects on S100β dynamics. Second, the study subgroups were of modest and unequal size (e.g., in the aSAH cohort n = 30 clipping, n = 20 coiling), reflecting real-world allocation patterns; this limits statistical power and may introduce imprecision in point estimates. Third, the serial measurement design involved multiple pairwise statistical comparisons across time points without formal correction for multiplicity; all time-course findings should be considered exploratory and require prospective replication. Fourth, functional outcome was assessed using the Glasgow Outcome Scale rather than the modified Rankin Scale: while GOS is well-established and widely used in the SAH literature, mRS provides finer gradation of functional disability and may offer greater sensitivity to detect differences in patients with moderate impairment. Finally, this is a single-center study with a defined enrollment window; the generalizability of the findings to other patient populations, treatment protocols, and assay platforms requires validation in external cohorts. Consequently, the present findings should be viewed as exploratory temporal biomarker data that require external validation before translation into clinical decision-making algorithms.

## 6 Conclusion

Acute-phase serum S100β elevations in this prospective aSAH cohort were closely linked to unfavorable six-month outcome, suggesting that S100β may represent a potentially useful candidate marker of early brain injury, disease severity and early risk stratification. Although early post-procedural S100β levels were higher after surgical clipping than after endovascular treatment, this transient difference resolved by approximately day 3 and did not translate into modality-specific outcome differences, indicating that early peaks primarily reflect overall injury burden and peri-interventional factors rather than durable procedural damage. By combining dense serial sampling in both ruptured and elective aneurysm patients with a standardized, modality-stratified analysis, this study provides a detailed temporal map of S100β dynamics after aSAH and emphasizes that the timing and trajectory of biomarker release are critical for interpretation, whereas isolated single time-point measurements are unlikely to support robust risk stratification.

## Declaration of competing interest

The authors declare that they have no known competing financial interests or personal relationships that could have appeared to influence the work reported in this paper.
